# Enhanced GABAergic Inputs Contribute to Functional Alterations of Cholinergic Interneurons in the R6/2 Mouse Model of Huntington’s Disease^1^,^2^,^3^

**DOI:** 10.1523/ENEURO.0008-14.2015

**Published:** 2015-03-23

**Authors:** Sandra M. Holley, Prasad R. Joshi, Anna Parievsky, Laurie Galvan, Jane Y. Chen, Yvette E. Fisher, My N. Huynh, Carlos Cepeda, Michael S. Levine

**Affiliations:** Intellectual and Developmental Disabilities Research Center, Semel Institute for Neuroscience and Human Behavior, University of California at Los Angeles, Los Angeles, California 90095

**Keywords:** cholinergic interneurons, GABA, Huntington’s disease, optogenetics, R6/2 mouse model, striatum

## Abstract

In Huntington’s disease (HD), a hereditary neurodegenerative disorder, striatal medium-sized spiny neurons undergo degenerative changes. In contrast, large cholinergic interneurons (LCIs) are relatively spared. However, their ability to release acetylcholine (ACh) is impaired. The present experiments examined morphological and electrophysiological properties of LCIs in the R6/2 mouse model of HD. R6/2 mice show a severe, rapidly progressing phenotype. Immunocytochemical analysis of choline acetyltransferase-positive striatal neurons showed that, although the total number of cells was not changed, somatic areas were significantly smaller in symptomatic R6/2 mice compared to wild-type (WT) littermates, For electrophysiology, brain slices were obtained from presymptomatic (3-4 weeks) and symptomatic (>8 weeks) R6/2 mice and their WT littermates. Striatal LCIs were identified by somatic size and spontaneous action potential firing in the cell-attached mode. Passive and active membrane properties of LCIs were similar in presymptomatic R6/2 and WT mice. In contrast, LCIs from symptomatic R6/2 animals displayed smaller membrane capacitance and higher input resistance, consistent with reduced somatic size. In addition, more LCIs from symptomatic mice displayed irregular firing patterns and bursts of action potentials. They also displayed a higher frequency of spontaneous GABAergic IPSCs and larger amplitude of electrically evoked IPSCs. Selective optogenetic stimulation of somatostatin- but not parvalbumin-containing interneurons also evoked larger amplitude IPSCs in LCIs from R6/2 mice. In contrast, glutamatergic spontaneous or evoked postsynaptic currents were not affected. Morphological and electrophysiological alterations, in conjunction with the presence of mutant huntingtin in LCIs, could explain impaired ACh release in HD mouse models.

## Abstract (precis)

Although large cholinergic interneurons (LCIs) in striatum are spared in Huntington's disease (HD), deficits in cholinergic function have been described. Here we demonstrate in a mouse model of HD that the firing patterns of LCIs are disrupted and this is due to aberrant GABAergic neurotransmission. This explains cholinergic deficits in HD.

## Significance Statement

Although large cholinergic interneurons (LCIs) in striatum are spared in Huntington's disease (HD), deficits in cholinergic function have been described. Here we demonstrate in a mouse model of HD that the firing patterns of LCIs are disrupted and this is due to aberrant GABAergic neurotransmission. This explains cholinergic deficits in HD.

## Introduction

Huntington’s disease (HD) is a fatal, hereditary, neurodegenerative disorder characterized by abnormal movements (chorea), cognitive impairment, and psychiatric disturbances (Bates et al., 2002). Histopathologically, there is progressive loss of cortical and striatal neurons with relative sparing of many (Ferrante et al., 1985; Ferrante et al., 1987; Reiner et al., 1988; Vonsattel and DiFiglia, 1998) but not all classes of striatal interneurons (Reiner et al., 2013). In the striatum, medium-sized spiny neurons (MSNs), the main projection neurons, undergo degenerative changes and, in animal models of HD, display a range of abnormal morphological and electrophysiological properties. These include decreased somatic areas (Levine et al., 1999) and loss of dendritic spines (Klapstein et al., 2001), as well as alterations in both passive and active membrane properties and synaptic activity (Klapstein et al., 2001; Cepeda et al., 2003; Cepeda et al., 2007).

Large cholinergic interneurons (LCIs) constitute a small percentage (∼2%) of striatal neurons but they strongly modulate the functional status of MSNs in physiological and pathological conditions (Pisani et al., 2007; Ding et al., 2010). Excitatory glutamatergic inputs to LCIs are mainly provided by the thalamus and less so by the cerebral cortex (Lapper and Bolam, 1992; Sadikot et al., 1992). MSNs and GABAergic interneurons provide inhibitory inputs to LCIs (Sullivan et al., 2008; Chuhma et al., 2011; Gonzales et al., 2013). Which properties or mechanisms endow LCIs with resistance to degeneration in HD remains unknown. One possibility is that LCIs receive only sparse cortical or thalamic inputs and NMDA receptor-mediated responses are significantly smaller compared with those in MSNs (Cepeda et al., 2001). Indeed, cell swelling (an early sign of excitotoxicity) induced by prolonged application of NMDA produces a time-dependent increase in somatic area in MSNs, whereas it produces negligible changes in LCIs (Cepeda et al., 2001). Another possibility is the fact that huntingtin nuclear inclusions form more gradually and are less abundant in LCIs compared with MSNs (Fusco et al., 1999; Kosinski et al., 1999; Meade et al., 2002).

Although spared in HD, LCIs display functional abnormalities, including impaired ability to release ACh (Vetter et al., 2003; Smith et al., 2006; Farrar et al., 2011) and defective synaptic plasticity (Picconi et al., 2006). In order to elucidate the role of LCIs in the progression of HD symptoms, we examined morphological and electrophysiological changes in LCIs in the R6/2 genetic mouse model of HD. R6/2 mice with ∼150 CAG (glutamine) repeats display a very aggressive form of the disease, more like juvenile HD (Mangiarini et al., 1996). They start to develop symptoms around 5-6 weeks of age and are fully symptomatic by 9-11 weeks. Using this model, a progressive reduction of glutamatergic inputs onto MSNs was demonstrated (Cepeda et al., 2003), whereas GABAergic inputs were increased (Cepeda et al., 2004; Cepeda et al., 2013). Similar changes also have been observed in other genetic mouse models of HD (Cummings et al., 2010). In the present study, morphological, electrophysiological, and optogenetic techniques were used to examine alterations of LCIs in the R6/2 model.

## Material and Methods

### Animals

All experimental procedures were performed in accordance with the United States Public Health Service Guide for Care and Use of Laboratory Animals and were approved by the Institutional Animal Care and Use Committee at the University of California, Los Angeles (UCLA). Every effort was made to minimize pain and discomfort. R6/2 transgenic mice and wild-type (WT) littermates were obtained from the breeding colony in the institutional vivarium. These mice were maintained by crossing WT male C57BL/6xCBA mice with WT female C57BL/6xCBA mice that had transplanted R6/2 ovaries (both males and females purchased from The Jackson Laboratory). Genotyping was performed using PCR of DNA obtained from tail samples, once at weaning and again following use for experimentation to confirm the genotype. R6/2 mice (∼150-160 repeat length) were utilized at presymptomatic (3-4 weeks) and symptomatic (>8 weeks) stages and compared with their WT littermates. For optogenetic experiments, somatostatin (SOM)-expressing interneurons and parvalbumin (PV)-expressing interneurons in WT and R6/2 mice were targeted by crossing homozygote male SOM-IRES-Cre mice (Sst^tm2.1(cre)Zjh^/J) or male PV-IRES-Cre (B6.129P2-Pvalb^tm1(cre)Arbr^/J) mice crossed with WT female C57BL/6xCBA mice transplanted with R6/2 ovaries (The Jackson Laboratory).

### ChAT immunostaining and cell counting

Experimenters blind to animal genotype performed these measurements. LCI somatic area measurements were obtained from symptomatic R6/2 mice and their WT littermates. Mice were perfused intracardially with 4% PFA in 0.1 M PBS. Brains were then extracted and immersed in the same solution overnight. An antibody against choline acetyltransferase (ChAT) was utilized to visualize the LCIs in the striatum. Slices from WT and their R6/2 littermates were processed in tandem. Mouse brains were cryoprotected first in 20% sucrose/0.1 M PBS solution then 30% sucrose/0.1 M PBS solutions overnight and frozen with powdered dry ice. Coronal slices (30 μm) containing the striatum from each mouse were prepared using a Microm HM 505E cryostat and collected in wells containing 0.5 M Tris-buffered saline (TBS, pH 7.4). Slices were then placed in a 10% methanol with 10% of 30% hydrogen peroxide TBS solution for 5 min to block endogenous peroxidases. After three washes (5 min each) in 0.05% Triton-TBS (pH 7.4), slices were blocked in 1.5% normal rabbit serum (Vector S-5000) for 1 h and then placed in goat anti-ChAT (Millipore, catalog number AB144P, 1:100) overnight at 4 °C. After another set of three washes (5 min each) in 0.05% Triton-TBS, slices were placed in rabbit anti-goat (Vector BA-5000, 1:300) for 35 min at room temperature. Slices were washed three times in 0.05% Triton-TBS for 5 min each and placed in Avidin Biotin Peroxidase Complex (Vector PK-6100) for 1 h followed by three more 5 min washes in 0.05% Triton-TBS. Slices were developed in diaminobenzidine, washed in cold TBS, mounted onto slides, and coverslipped with Cytoseal XYL Mounting Media.

Stereological methods (Gundersen and Jensen, 1987) were used to determine striatal volume (excluding the nucleus accumbens). First, all slices containing the striatum [27 hemi-slices, thickness (*H*) = 30 µm] were imaged using a 20× objective on a Zeiss microscope (Axio Imager.M2) and an AxioCam MR3 camera. All ChAT+ neurons were counted in each hemi-slice and two were randomly selected for somatic area measurements. Light homogeneity was ensured after adjusting the white balance and all images were acquired using the same light threshold. Measurements were obtained using the Zeiss AxioVision software (version 4.8.2 SP3). Volume estimates were obtained based on Cavalieri’s principle using the following formula: Volume = d*(*a*1 + *a*2 + *a*3 + *a*4 …), where *d* is the distance between serial sections (30 µm), and *a* is the area containing ChAT+ cells. ChAT+ cells as well as their diameters (*h*) were determined using the same software. For each slice, the ChAT+ cell number was corrected by the Abercrombie factor (*N*, where *N* = *h*/(*h* + *H*)). Then, the density (ChAT+ cells/mm^3^) was obtained by dividing the corrected number of ChAT+ cells by the Cavalieri’s striatal volume (mm^3^).

### Electrophysiology in brain slices

Mice were deeply anesthetized with halothane or isoflurane and decapitated. The brains were dissected and immediately placed in oxygenated ice-cold low-Ca^2+^ artificial CSF (ACSF) containing (in mM): 130 NaCl, 3 KCl, 1.25 NaH_2_PO_4_, 26 NaHCO_3_, 5 MgCl_2_, 1 CaCl_2_, and 10 glucose. Coronal slices (300 μm) were cut and transferred to an incubating chamber containing ACSF (with 2 mm CaCl_2_ and 2 mm MgCl_2_) oxygenated with 95% O_2_-5% CO_2_ (pH 7.2-7.4, osmolality 290-310 mOsm, 25 ± 2 °C). A single slice was then transferred to a submerged recording chamber fixed to an upright microscope. LCIs were visualized in the dorsolateral striatum using infrared illumination with differential interference contrast optics (IR-DIC microscopy) and identified based on large somatic size (at least twice the size of a typical MSN), basic membrane properties, as well as their characteristic tonic action potential firing observed in the cell-attached mode.

The patch pipette (series resistance 3-5 MΩ) contained the following solution (in mM): 130 Cs-methanesulfonate, 10 CsCl, 4 NaCl, 1 MgCl_2_, 5 MgATP, 5 EGTA, 10 HEPES, 0.5 GTP, 10 phosphocreatine, 0.1 leupeptin (pH 7.25-7.3, osmolality, 280-290 mOsm) (Cs-Meth solution). In a subset of experiments, CsCl-based internal solution with a higher concentration of Cl^−^ ions (140 mM) was used (CsCl solution). Access resistances were <20 MΩ and all the recordings were made at ambient temperature (22-26 °C).

To examine firing patterns and pacemaking of LCIs, 2-5 min periods of spontaneous activity in the cell-attached configuration were analyzed. The Mini Analysis Program (Synaptosoft) was used to calculate interspike intervals (ISI) from which the degree of autocorrelation and coefficient of variation can be derived. Degree of autocorrelation was defined as a function of the coefficient value (peak amplitude of the first lag period) and the number of peaks away from lag 0. Autocorrelations were assigned high or low ratings based on correlation coefficients and visual inspection of the histograms from each cell. A histogram with high autocorrelation had correlation coefficients >0.2 (first peak value range 0.203–0.8) and showed additional peaks at different lag periods, while a low autocorrelation had correlation coefficients <0.2 (first peak value range 0.08–0.2) and showed only 1–2 peaks. Cells with no correlation showed no peaks except at the origin (lag 0) where the correlation is always 1 (this peak was eliminated from the autocorrelograms). This criterion to define cells with high or low degree of autocorrelation was used in a previous publication (Cepeda et al., 2014). The coefficient of variation, a common measure of dispersion of a probability distribution, was defined as the ratio of the standard deviation (SD) to the calculated mean ISI (Bennett and Wilson, 1999). LCIs with high coefficient of variation had low degree of autocorrelation and vice versa.

Spontaneous postsynaptic currents were recorded in the whole-cell configuration in standard ACSF and in the presence of appropriate receptor blockers. Membrane current was filtered at 1 kHz and digitized at 200 μs using Clampex 8.2 (gap-free mode). Cells were voltage-clamped at −70 mV to determine basic membrane properties using a depolarizing voltage command (10 mV). Glutamate receptor mediated spontaneous EPSCs (sEPSCs) were recorded in the presence of the GABA_A_ receptor blocker, bicuculline (BIC, 10 μM). As the frequency of spontaneous EPSCs is generally very low in LCIs, in another set of experiments 4-aminopyridine (4-AP, a selective K^+^-channel blocker) was coapplied (100 μM) to enhance neurotransmitter release and increase the frequency and amplitude of sEPSCs. Application of the AMPA receptor blocker, 2,3-dihydroxy-6-nitro-7-sulfamoyl-benzo[f]quinoxaline-2,3-dione (NBQX, 10 μM) and the NMDA receptor blocker amino-5-phosphonovaleric acid (APV, 50 μM) abolished all spontaneous EPSC activity (data not shown). Evoked glutamatergic EPSCs (eEPSCs) were recorded at −70 mV. Lidocaine (QX314, 4 mM) was added to the internal pipette solution to block Na^+^ channel-mediated currents. To evoke synaptic currents in LCIs, a monopolar glass stimulating-electrode (patch-pipette filled with ACSF) was placed in the striatum approximately 150-200 μm dorsal to the recorded cell. Test stimuli (0.5 ms duration) were applied every 20 s and responses averaged over two to three consecutive trials. Test stimuli were applied at increasing stimulus intensities (0.01–0.10 mA) to assess input–output functions. These recordings were obtained in the presence of BIC (10 μM). Coapplication of NBQX (10 μM) and APV (50 μM) completely blocked the evoked currents.

To assess GABA_A_ receptor-mediated spontaneous synaptic activity, cells were voltage clamped at +20 mV and spontaneous IPSCs (sIPSCs) were recorded in the presence of ionotropic glutamate receptor blockers (10 μM NBQX and 50 μM APV). Alternatively, sIPSCs also were recorded with cells voltage clamped at −70 mV with the pipette solution containing a high concentration (140 mM) of Cl^−^ ions. Tetrodotoxin (TTX, 1 μM) was added to record miniature IPSCs (mIPSCs). Evoked IPSCs (eIPSCs) were recorded at +10 mV, using the Cs-Meth internal solution or at −70 mV using the high Cl^−^ solution. QX314 was added to the patch pipette solution to block Na^+^ channel-mediated currents. These recordings were obtained in the presence of ionotropic glutamate receptor blockers, as indicated above. In both sets of experiments, BIC (10 μM) blocked spontaneous or eIPSCs (data not shown). Spontaneous EPSCs and IPSCs were analyzed offline using the Mini Analysis Program. Evoked synaptic currents were analyzed using pClamp 8.2 or 10.2 (Molecular Devices).

### AAV stereotaxic injections and optical stimulation

Stereotaxic injections of channelrhodopsin-2 (ChR2) in SOM-Cre and PV-Cre mice crossed with R6/2 or WTs were performed at 4-5 weeks of age. Mice were anesthetized with isoflurane and injected with an adeno-associated virus (AAV2) expressing DIO-channelrhodopsin-2 (ChR2(H134R)) and enhanced yellow fluorescent protein (EYFP). This construct contained a floxed stop codon and the EF1a promoter was used for protein expression (Cardin et al., 2009; Sohal et al., 2009). One microliter of virus (titer: 9.45 × 10^12^ vg/ml) was injected into each striatum at a rate of 0.2 μl/min and at the following stereotaxic coordinates from bregma: AP 1.0 mm, ML +/−2.0 mm, and DV −3.3 mm from the dura. For the thalamus in R6/2 and WT mice, an AAV2 construct with ChR2(H134R)-EYFP under the Ca^2+^/calmodulin-dependent protein kinase type II alpha subunit (CaMKIIa) promoter (titer: 1.14 × 10^13^ vg/ml) was injected. Bilateral injections (0.25 μl) were made in the centromedian-parafascicular (Cm/Pf) intralaminar nuclear complex of the thalamus (AP −2.2 mm, ML +/− 0.7 mm, DV −3.5 mm) at a rate of 0.1 μl/min. All ChR2 plasmids were obtained from Karl Deisseroth (Stanford University, CA) and AAV production was performed at the University of Iowa Gene Transfer Vector Core.

In order to ensure sufficient expression of injected AAVs, recordings were performed at least 5-9 weeks following injections. PV- and SOM-expressing interneurons were activated with a single light pulse (470 nm, 0.5 ms, 10 mW, CoolLED) delivered through the epifluorescence illumination pathway using Chroma Technologies filter cubes. eIPSCs were recorded in LCIs in voltage-clamp mode, with a Cs-Meth-based internal solution at a holding potential of +10 mV and in the presence of glutamatergic receptor antagonists. In these experiments, biocytin (0.2%) was also included in the patch pipette to locate recorded LCIs.

For thalamic stimulation, excitatory responses were evoked by a brief light pulse (470 nm, 1 ms duration, 5 mW) in the presence of 10 µM BIC. AMPA currents were recorded in LCIs at a holding potential of −70 mV. NMDA currents were recorded at a holding potential of +40 mV in the presence of BIC and NBQX (10 μM). Patch pipettes were filled with Cs-Meth solution containing 4 mM QX-314 and 0.2% biocytin. To prevent stimulation of thalamocortical inputs, the cerebral cortex was removed from the slice before recording.

### Electrophysiology in acutely isolated neurons

Some slices also were used to record from acutely isolated large (presumably cholinergic) neurons. With the aid of a dissecting microscope, the dorsal striatum was dissected and incubated for at least 1 h at room temperature in an NaHCO_3_-buffered Earle’s balanced salts solution (Sigma-Aldrich) supplemented with (in mM): 1 pyruvic acid, 0.005 glutathione, 0.1 NG-nitro-L-arginine, and 1 kynurenic acid (pH 7.4; aerated with 95% O_2_/5% CO_2_; 300-310 mOsm/l). After incubation, a slice was placed in an oxygenated cell-stir chamber (Wheaton) and enzymatically treated for 20 min with papain (0.5 mg/ml; Calbiochem) at 35 °C in a HEPES-buffered HBSS (Sigma-Aldrich) and supplemented as described above. After enzymatic digestion, the tissue was rinsed with low-Ca^2+^ HEPES-buffered Na^+^ isethionate solution containing (in mM): 140 Na^+^ isethionate, 2 KCl, 2 MgCl_2_, 0.1 CaCl_2_, 23 glucose, and 15 HEPES (pH 7.4, 300-310 mOsm/l). Striatal slices were then mechanically dissociated with a series of graded fire-polished Pasteur pipettes. The cell suspension was then plated into a 35 mm Nunclon Petri dish containing a HEPES-buffered salt solution (in mM: 140 NaCl, 23 glucose, 15 HEPES, 2 KCl, 2 MgCl_2_, and 1 CaCl_2_; pH 7.4, 300-310 mOsm/l) and mounted on the stage of an upright fixed-stage microscope (Zeiss Axioscope).

The whole-cell patch-clamp technique in voltage clamp mode was used for recordings. Large neurons were identified based on somatic size (at least twice the size compared to MSNs). Signals were detected with an Axoclamp 2B amplifier (Axon Instruments). Glass pipettes (2.5-3.5 MΩ) were filled with an internal solution consisting of (in mM): 175 N-methyl-D-glucamine, 40 HEPES, 2 MgCl_2_, 10 EGTA, 12 phosphocreatine, 2 Na_2_ATP, 0.2 Na_2_GTP and 0.1 leupeptin (pH 7.25, 265-270 mOsm/l). The Mg^2+^-free external solution consisted of the following (in mM): 135 NaCl, 20 CsCl, 3 BaCl_2_, 2 CaCl_2_, 10 glucose, 10 HEPES, 0.02 glycine, and 0.0003 TTX (pH 7.4, 300-310 mOsm/l). After obtaining a GΩ seal and rupturing the membrane to create the whole-cell configuration, access resistances were <20 MΩ. Series resistance was then compensated (70-90%) and monitored throughout the experiment.

Drugs were applied through an array of capillaries positioned 500-600 μm from the cell, using a pressure-driven fast perfusion system. Solution changes were performed by changing the position of the array with a DC drive system controlled by a SF-77B perfusion system (Warner Instruments). Drugs were applied for 3 s every 30 s. Values for peak currents and current densities were calculated for all neurons. Current densities were calculated by dividing the peak current by the cell capacitance and currents were normalized to cell size.

### Statistics

Differences between group means were assessed with appropriate *t* tests (paired or unpaired) or Mann−Whitney rank sum test, chi-square or Fisher exact tests for proportions, and appropriately designed analyses of variance (two-way ANOVA with or without repeated measures, followed by Bonferroni’s or Tukey’s *post hoc* tests). Values in figures and text are presented as mean ± SEM. Differences were considered statistically significant if *p* < 0.05.

## Results

### LCI somatic areas are reduced in R6/2 mice

**Figure 1:**
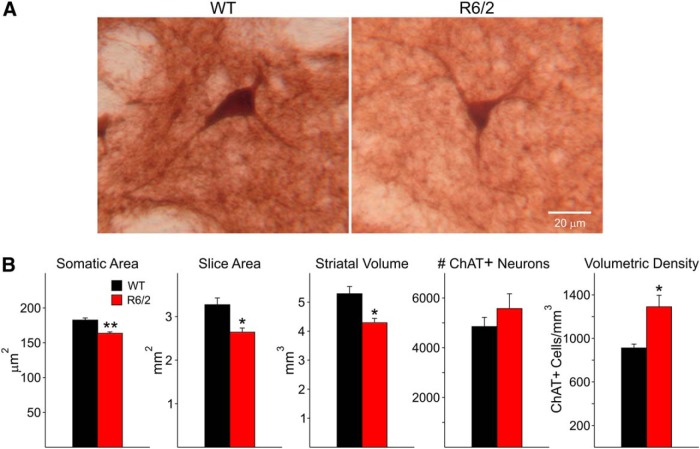
***A***, LCIs were stained with ChAT antibody in WT and symptomatic R6/2 mice. Less intense staining and reduced cell size were evident**. *B***, Bar graphs show that the mean somatic cross-sectional area was significantly smaller in R6/2 LCIs. In addition, mean striatal area was significantly reduced in hemi-slices from R6/2 mice. Striatal volume assessed stereologically was also significantly reduced. The number of striatal ChAT+ neurons was similar but, because of reduced striatal volume, the mean density of ChAT+ neurons was significantly increased in R6/2 mice. *p < 0.05, **p < 0.01.

Reduced MSN somatic area and dendritic elaboration have been previously shown in symptomatic R6/2 mice (Levine et al., 1999; Klapstein et al., 2001). However, morphological changes in LCIs have not been examined. We used ChAT immunohistochemistry to estimate cell numbers and changes in somatic area of LCIs in symptomatic R6/2 mice (*n* = 4, age 64 ± 2 d) and WT littermates (*n* = 4, age 60 ± 2 d). LCIs (*n* = 202 cells) from R6/2 mice showed a statistically significant decrease in somatic area compared to LCIs (*n* = 202 cells) from WT mice (*p* < 0.01; Fig. 1). To estimate changes in ChAT+ neuronal density, the area of individual slices was calculated first. Compared with WT littermates, mean striatal area of 30-μm-thick hemi-slices was significantly smaller in R6/2 compared to WT mice (*p* = 0.011). Stereological methods were used to calculate striatal volume and LCI total number and density. Striatal volume was significantly reduced in R6/2 compared to WT mice (*p* = 0.029), but the total numbers of ChAT+ neurons were not significantly different (*p* = 0.33). However, the stereological analysis revealed a statistically significant increase in striatal ChAT+ neuronal density (*p* = 0.013; Fig. 1*B*).

### Passive and active membrane properties of LCIs are altered in HD mice

**Figure 2:**
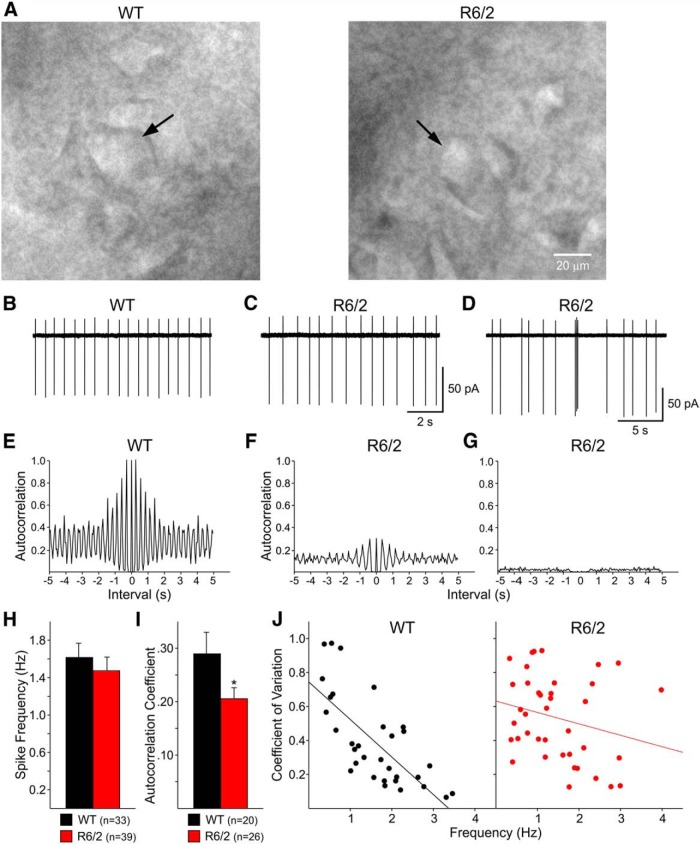
***A***, Infrared differential interference contrast optics was used to visualize LCIs (arrows) in WT and symptomatic R6/2 mice. ***B-D***, Representative traces of tonic firing of LCIs in the cell-attached configuration. In WTs, more LCIs fired action potentials regularly (***B***). In R6/2s, some LCIs fired regularly (***C***) but a greater number of irregular and bursting cells (***D***) were recorded. ***E***, High degree of autocorrelation reflected regular firing patterns of LCIs from WT mice. ***F***, Although firing of some LCIs from R6/2 animals was highly autocorrelated, the autocorrelation coefficient (peak amplitude or coefficient value of the first lag interval) was lower than in LCIs from WTs. ***G***, Low or no autocorrelations were observed in irregular firing and bursting LCIs, particularly from R6/2s. ***H***, Spontaneous firing rates in LCIs from WT and R6/2 mice were almost identical. ***I***, In contrast, the autocorrelation coefficient, a measure of firing regularity, was significantly decreased in LCIs from R6/2 mice. ***J***, Scatter plots showing the correlation between firing frequency and coefficient of variation. There was a very good correlation between both in LCIs from WT mice. In contrast, this correlation was markedly reduced in cells from R6/2 mice. *p < 0.05.

For electrophysiological studies LCIs were recorded primarily from the dorsolateral striatum. Representative IR-DIC images of visually identified LCIs from WT and R6/2 mice are shown in Fig. 2*A*. In whole-cell voltage-clamp mode, passive membrane properties were consistent with LCIs, displaying relatively high input resistance and fast decay time constant. No differences in membrane properties of LCIs from WT and presymptomatic R6/2 animals (*n* = 4 in each group, age 23 ± 1 d) were observed (Table 1). In symptomatic animals (*n* = 10, age 65 ± 1 d), LCIs displayed significantly lower mean capacitance (*p* = 0.038) and higher mean input resistance (*p* < 0.001) compared with WT animals (*n* = 14, age 65 ± 1 d).

**Table 1:** Passive membrane properties of LCIs in R6/2 mice

	Cm (pF)	Rm (MΩ)	Tau (ms)
Presymptomatic			
WT (*n* = 10)	89.5 ± 7	151 ± 22	1.2 ± 0.2
R6/2 (*n* = 12)	81.4 ± 5	177 ± 28	0.9 ± 0.1
Symptomatic			
WT (*n* = 31)	89.6 ± 4	198 ± 15	1.3 ± 0.1
R6/2 (*n* = 26)	80.0 ± 3*	335 ± 23***	1.2 ± 0.1

* *p* < 0.05,

*** *p* < 0.001.

Most LCIs from WT and R6/2 mice generated spontaneous action potentials in the cell-attached configuration at frequencies ranging between 0.3-3.9 Hz. To calculate the mean firing frequency, cell-attached recordings were obtained over a 2-5 min period. No differences were seen in LCI firing rates from presymptomatic R6/2 mice and WT littermates (data not shown). Mean firing frequencies (Fig. 2*H*) also were similar in LCIs from symptomatic R6/2 mice (Fig. 2*C*) compared with WTs (Fig. 2*B*). However, more LCIs from R6/2 mice displayed irregular firing patterns and bursts (Fig. 2*D*). Autocorrelation analysis of LCI firing demonstrated that about twice as many cells from WTs (10/33, 30.3%) displayed a high degree of autocorrelation compared with LCIs from R6/2s (5/39, 12.8%). To obtain a quantitative estimate of firing regularity, in a subset of cells (those that displayed some degree of autocorrelation, *n* = 20 WT and *n* = 26 R6/2) the coefficient value of the first lag period (adjacent to lag 0) was calculated using autocorrelation histograms (Fig. 2*E−G*). The average values were significantly higher in cells from WT compared to cells from R6/2 mice (0.29 ± 0.04 and 0.21 ± 0.02, respectively; *p* = 0.046; Fig. 2*I*), suggestive of decreased firing regularity in LCIs from R6/2 mice.

Another way to examine firing regularity is to compute coefficients of variation: the lower the coefficient, the higher the regularity. Using 0.2 as the cutoff value to match the average autocorrelation coefficients, more cells from WTs (11/33, 33.3%) had low coefficients of variation (<0.2) compared with those from R6/2s (4/39, 10.2%). The difference between groups was statistically significant (*p* = 0.02). Finally, scatter plots demonstrated a strong correlation between coefficients of variation and firing frequency in LCIs from WT mice (linear regression, *r* ^2^ = 0.52, *p* < 0.001), whereas in cells from R6/2 mice, this correlation was greatly reduced (*r* ^2^ = 0.06, *p* = 0.14; Fig. 2*J*). Similarly, the Pearson product moment correlation demonstrated a very high correlation in LCIs from WTs (−0.72, *p* < 0.0001), whereas the correlation was not statistically significant in cells from R6/2 mice (−0.24, *p* = 0.14). Together, these data provide evidence that the ability of LCIs to regularly generate action potentials becomes compromised in symptomatic mice, suggesting alterations in intrinsic conductances and/or synaptic inputs.

### Spontaneous GABA_A_ receptor-mediated synaptic currents are increased in LCIs from symptomatic R6/2 mice

**Figure 3:**
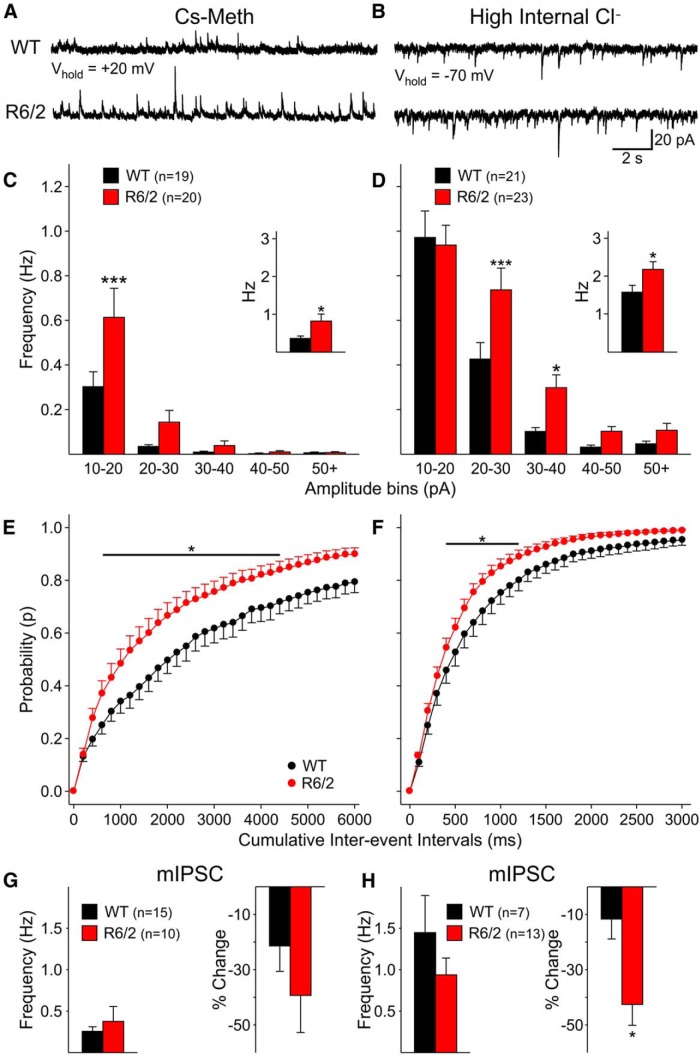
***A***, ***B***, Representative traces of spontaneous IPSCs in LCIs from R6/2 and WT mice at 65 d using Cs-Meth (***A***) or CsCl (***B***) internal solutions. In both cases, spontaneous IPSCs occurred more frequently in LCIs from R6/2 mice. ***C***, ***D***, Amplitude−frequency histograms of sIPSCs using Cs-Meth (***C***) and CsCl (***D***) internal solutions. In most amplitude bins, there was an increase in frequency in LCIs from R6/2 mice. The insets show the mean sIPSC frequency. Regardless of the internal solution used, there was a significant increase in LCIs frequency in R6/2s. ***E***, ***F***, Cumulative probability histograms of interevent intervals in WT and R6/2 mice at 65 d using Cs-Meth (***E***) and CsCl (***F***) internal solutions. A significant increase in short interevent intervals was observed in LCIs from R6/2 mice. ***G***, ***H***, Average frequency of mIPSCs in LCIs from WT and R6/2 mice was similar using Cs-Meth (***G***) or CsCl (***H***) internal solutions. The percent reduction in IPSC frequency after TTX was greater in LCIs from R6/2 mice and this difference was statistically significant for the CsCl internal solution (***H***, right). *p < 0.05, ***p < 0.001.

MSNs from symptomatic R6/2 mice receive increased GABAergic inputs and feedforward inhibitory circuits play a major role in this effect (Cepeda et al., 2013). To determine if LCIs also are subject to increased GABAergic inhibition, sIPSCs were recorded (in the presence of ionotropic glutamate receptor antagonists) using the Cs-Meth internal solution and cells were held at +20 mV to increase the driving force of Cl^−^ ions. The frequency of sIPSCs (1.7 ± 0.3 Hz in WT vs 1.1 ± 0.6 Hz in R6/2, *p* = 0.42) was not significantly different in LCIs from presymptomatic R6/2 compared to WT mice (*n* = 4 in each group, age 23 ± 1 d). In contrast, in symptomatic R6/2 mice (*n* = 10, age 64 ± 1 d), the average frequency of sIPSCs was significantly increased compared with WTs (*n* = 12, age 65 ± 1 d; Fig. 3*A* and *C*, inset). This increase was observed across amplitude bins and was statistically significant for the 10-20 pA bin (Fig. 3*C*). Further, the cumulative interevent interval probability distribution was shifted to the left, indicating more short interevent intervals in cells from R6/2 mice (*p* < 0.001; Fig. 3*E*). Application of TTX (1 μM) eliminated the increase in sIPSC frequency (Fig. 3*D*), indicating its dependence on presynaptic action potentials. In addition, the percent reduction in IPSC frequency after TTX application was greater in cells from R6/2 compared with WT mice, suggesting that more sIPSCs from R6/2 cells were action potential-dependent (Fig. 3*G*, right bar graph).

We also examined the frequency of GABAergic sIPSCs in WT (*n* = 12, age 65 ± 1 d) and symptomatic R6/2 animals (*n* = 13, age 64 ± 2 d) using a CsCl-based internal solution with the cell voltage-clamped at −70 mV and in the presence of glutamate receptor blockers. Mean sIPSC frequency was significantly greater in LCIs from R6/2 mice (*p* < 0.01; Fig. 3*B* and *D*, inset). The amplitude-frequency histogram showed that, except for the 10-20 pA bin, increased frequency occurred across all other amplitude bins (Fig. 3*D*), in particular IPSCs with amplitudes between 20-40 pA (*p* < 0.001). The cumulative interevent interval probability distribution also was significantly shifted to the left in LCIs from R6/2 mice (*p* < 0.001; Fig. 3*F*). Similar to results obtained with Cs-Meth solution, the frequency of mIPSCs in LCIs recorded with CsCl after TTX were not significantly different between WTs and R6/2s (Fig. 3*H*). The percent change in frequency after TTX was significantly reduced in LCIs from R6/2s (*p* = 0.02; Fig. 3*H*, right bar graph), indicating that more sIPSCs were dependent on presynaptic action potentials.

### Evoked GABAergic currents are increased in LCIs from symptomatic R6/2 mice

**Figure 4:**
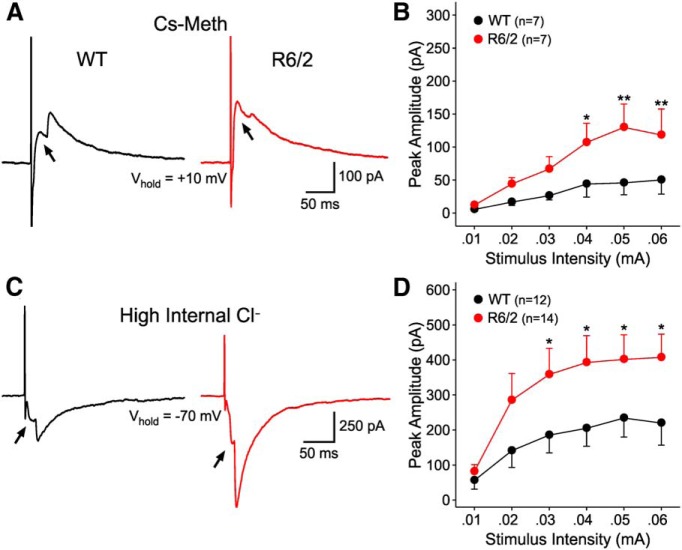
***A***, Representative traces of eIPSCs recorded with Cs-Meth internal solution and holding the membrane at +10 mV in LCIs from WT and symptomatic R6/2 mice. eIPSCs had multiple components and amplitude of the earliest peak was measured (arrows). ***B***, Graph shows the input−output relationship. Current response amplitudes were significantly increased at intensities of 0.04 mA and higher. ***C***, eIPSCs recorded in LCIs at −70 mV with high Cl^−^ internal solution in WT and R6/2 mice at 65 d. ***D***, Similar to recordings with Cs-Meth, there was a significant increase in IPSC amplitudes in cells from R6/2 mice. *p < 0.05, **p < 0.01.

eIPSCs were recorded at +10 mV in the presence of glutamate receptor antagonists. Intrastriatal stimulation typically evoked polysynaptic GABAergic currents with two or more peaks (Fig. 4*A*,*C*). Application of BIC (10 μM) completely blocked all evoked currents. Amplitude of peak current with the shortest latency, presumably monosynaptic, was measured (arrows in Fig. 4*A*,*C*). eIPSC peak currents were similar over a range of stimulus intensities (0.01-0.06 mA) in LCIs from WT and presymptomatic R6/2 mice (data not shown). In LCIs from symptomatic animals (*n* = 3, age 61 ± 3 d), mean peak currents recorded at +10 mV holding potential using the Cs-Meth internal solution were significantly larger (*p* < 0.001) than in LCIs from WT mice (*n* = 3, age 66 ± 5 d; Fig. 4*B*). Evoked GABAergic currents also were examined using high Cl^−^ internal solution, with cells voltage-clamped at −70 mV. Under these conditions, intrastriatal stimulation also typically evoked polysynaptic inward currents with characteristic multiple peaks (Fig. 4*C*). Amplitudes of peak currents with the shortest latency were significantly greater across a range of stimulus intensities in LCIs from R6/2 compared with mice (*p* < 0.001; Fig. 4*D*).

To determine the possible source of increased GABA responses at the presynaptic or postsynaptic sites, paired-pulse ratios (PPRs) were calculated for eIPSCs at different interpulse intervals (25, 50, and 100 ms). The differences in PPRs were not statistically significant in LCIs from R6/2 mice (at 25 ms interval, 1.22 ± 0.4 and 1.07 ± 0.3; at 50 ms interval, 1.26 ± 0.8 and 1.18 ± 0.7; at 100 ms interval, 1.01 ± 0.5 and 0.85 ± 0.4 in WT and R6/2, respectively).

### LCI responses to optogenetic activation of SOM- but not PV-expressing interneurons are increased in HD compared to WT mice

**Figure 5:**
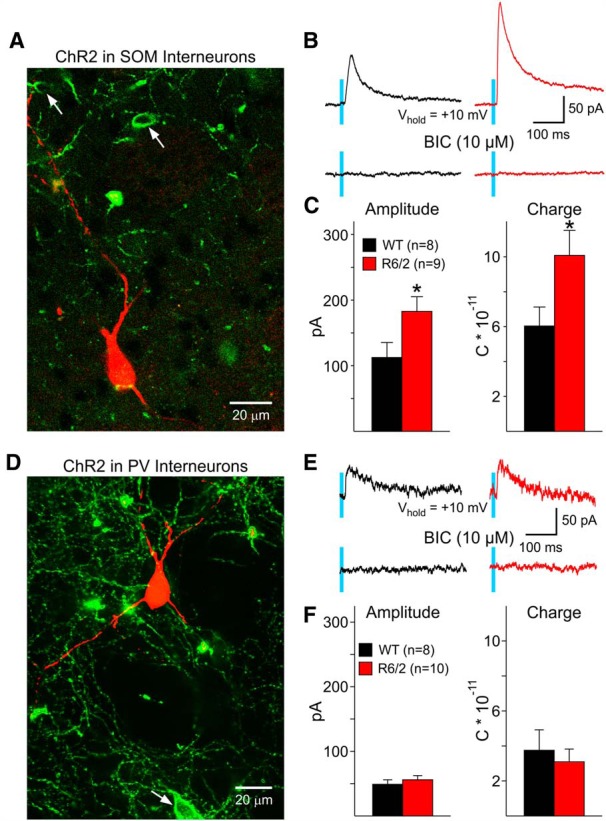
***A***, Confocal image (*z*-stack) of a LCI recorded and filled with biocytin (red). The LCI is surrounded by ChR2-EYFP terminals from SOM interneurons (green). Arrows indicate SOM-expressing interneurons. ***B***, IPSCs evoked in LCIs by optogenetic stimulation of SOM-expressing interneurons. Responses were significantly larger in LCIs from R6/2 mice. These responses were completely blocked by BIC. ***C***, Graphs show significant increases in amplitude and charge in LCIs from R6/2 mice compared to WTs. ***D***, LCI surrounded by ChR2-EYFP terminals from PV interneurons. Arrow indicates a PV-expressing interneuron. ***E***, IPSCs evoked in LCIs by optogenetic stimulation of PV-expressing interneurons were smaller than those evoked by SOM terminal stimulation and were not different between WT and R6/2 animals. Responses were completely blocked by BIC. ***F***, Graphs show the lack of significant differences in peak amplitude and charge in LCIs from WT and R6/2 mice. *p < 0.05.

Feedforward inhibition, in particular from SOM-expressing persistent low-threshold spiking (PLTS) interneurons, plays a crucial role in the increased GABA synaptic activity observed in MSNs from R6/2 mice (Cepeda et al., 2013). To determine if SOM-expressing PLTS interneurons serve as a source for increased GABA synaptic activity in LCIs, we used an optogenetic approach to isolate the contribution of these interneurons (Fig. 5*A*). LCIs were voltage clamped at +10 mV and IPSCs were evoked by optically activating ChR2 expressed in SOM interneurons (Fig. 5*B*, top). In LCIs from WT mice (*n* = 7, age 71 ± 2 d), 62% of cells responded to SOM interneuron activation (8/13) and in LCIs from R6/2 mice (*n* = 6, age 70 ± 1 d), 82% of cells responded (9/11 cells) (*p* = 0.27). The eIPSCs in LCIs from R6/2 mice had significantly larger peak amplitudes (*p* < 0.05), greater charge (*p* < 0.05 pA) (Fig. 5*C*), and longer decay times (125 ± 8 ms vs 98 ± 10 ms, *p* < 0.05, for R6/2 and WT, respectively) when compared to responses recorded in WT LCIs. All eIPSCs were identified as GABAergic, as all recordings were performed in the presence of NBQX and APV (10 μM and 50 μM, respectively), and responses were completely eliminated after addition of BIC (10 μM) (Fig. 5*B*, bottom).

We also examined the effects of optical activation of PV-expressing, fast-spiking interneurons (FSIs) in symptomatic R6/2 (*n* = 6, age 74 ± 1 d) and WT littermates (*n* = 7, age 78 ± 3 d). PV interneuron activation produced few IPSC responses in LCIs from R6/2 (10/16, 59%) and WT (8/21, 38%) mice (*p* = 0.19) and the amplitude of the response was much smaller compared with responses evoked by SOM-expressing interneurons (Fig. 5*D*,*E*). When comparing the evoked responses, there was no difference in the peak amplitude, charge (Fig. 5*F*), or decay times (R6/2: 74 ± 16 ms vs WT: 86 ± 26 ms) in LCIs from R6/2 compared to WT. These results suggest that SOM-positive PLTS interneurons, and not PV-positive FSIs, contribute to the increased GABAergic inputs to LCIs of symptomatic R6/2 mice. Although it could be argued that the small and inconsistent GABA responses evoked by PV terminals in LCIs could be explained by insufficient expression of ChR2, this is unlikely, as previous studies using similar Cre lines demonstrated large responses to PV activation in MSNs (Cepeda et al., 2013). This was corroborated in the present study by recording consistent, large-amplitude GABA responses in a small number of MSNs adjacent to LCIs (data not shown).

### Spontaneous and evoked glutamatergic currents

Excitatory synaptic inputs also could alter tonic firing patterns of LCIs. Spontaneous glutamatergic EPSCs were measured in LCIs, with cells voltage-clamped at −70 mV, in the presence of BIC (10 μM). Consistent with previous studies (Bennett and Wilson, 1999), very few sEPSCs were observed under these conditions. Subsequently, the frequency of sEPSCs was enhanced by coapplication of 4-AP (100 μM), a K^+^ channel blocker that enhances neurotransmitter release. Average sEPSC frequencies (7.8 ± 3.4 Hz, *n* = 6 WT and 4.4 ± 1.4 Hz, *n* = 6 R6/2, *p* = 0.3), amplitude-frequency histograms, and cumulative probability of interevent intervals were not significantly different between LCIs from R6/2 mice (*n* = 4, age 76 ± 6 d) and their WT littermates (*n* = 4, age 74 ± 6 d) (data not shown).

**Figure 6:**
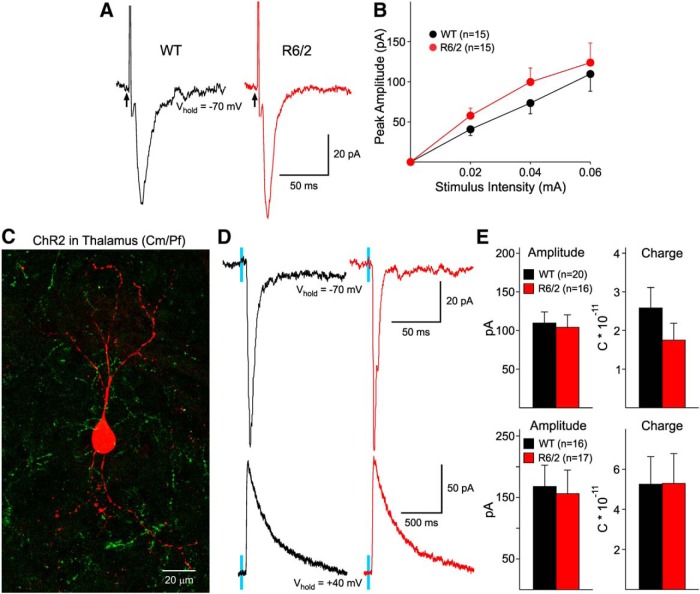
***A***, Representative traces of eEPSCs in LCIs recorded at −70 mV using Cs-Meth internal solution. The peak amplitude of the response was similar in LCIs from WT and R6/2 mice. ***B***, Graph shows the input−output relationship of eEPSCs. No statistical differences in amplitude were observed. ***C***, Striatal LCI (red) surrounded by EYFP terminals in a R6/2 mouse injected with ChR2 in the Cm/Pf nuclear complex. ***D***, Optogenetic stimulation of thalamic afferents induced similar AMPA (top) and NMDA (bottom) receptor-mediated currents in LCIs from WT and R6/2 mice. ***E***, Graphs show that the amplitude and charge of AMPA (top) and NMDA (bottom) responses were not significantly different.

eEPSCs were recorded at −70 mV with QX314 in the internal pipette solution (Fig. 6*A*). Similar procedures to those described above were used to evoke synaptic currents in LCIs. Application of NBQX (10 μM) and APV (50 μM) completely blocked evoked currents. There were no statistically significant differences in peak amplitudes of LCIs from symptomatic R6/2 (*n* = 8, age 65 ± 1 d) compared with those from WTs (*n* = 6, age 66 ± 2 d) at all stimulation intensities (Fig. 6*A*,*B*).

### LCI responses to optogenetic activation of thalamic inputs are similar in WT and R6/2 mice

The main excitatory input onto LCIs has been shown to emanate from the thalamus (Lapper and Bolam, 1992). Expression of ChR2 in the Cm/Pf nuclear complex allows stimulation of specific thalamostriatal projections onto LCIs. These projections are glutamatergic and when stimulated result in eEPSCs. At a holding potential of −70 mV, in the presence of BIC, the eEPSC is attributable to activation of AMPA receptors (Fig. 6*D*, top). Amplitude and charge of AMPA currents in R6/2 mice (*n* = 12, age 75 ± 2 d) were similar to those from WTs (*n* = 13, age 79 ± 2 d) (Fig. 6*D*,*E*). Decay times also were similar (37 ± 7 ms in WT and 23 ± 4 ms in R6/2). At a holding potential of +40 mV, in the presence of BIC and NBQX, the eEPSC is attributed to currents through activation of NMDA receptors (Fig. 6*D*, bottom). NMDA currents showed no differences in peak amplitude, charge, or kinetics between WT and R6/2 mice (Fig. 6*E*).

### Glutamate and GABA responses in acutely dissociated LCIs are similar in WT and R6/2 mice

**Figure 7:**
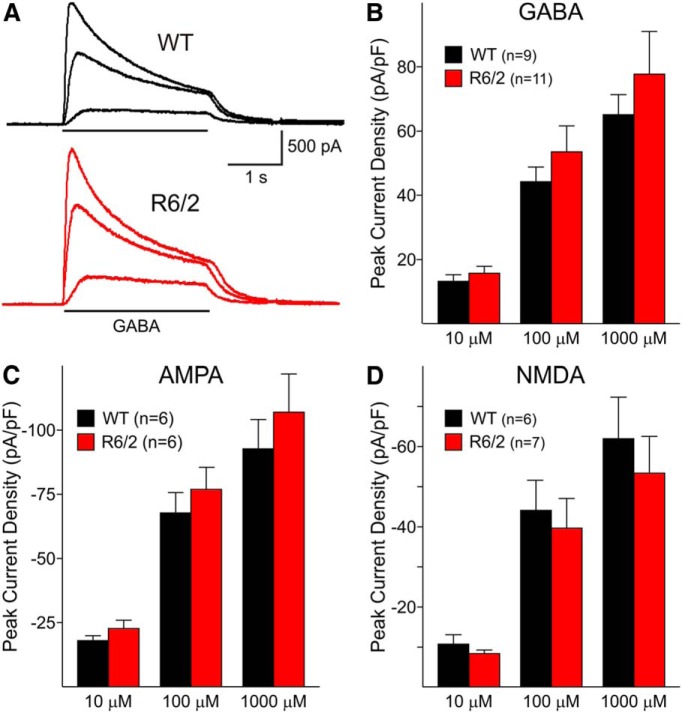
***A***, Representative traces of responses induced by bath application of increasing concentrations of GABA (10, 100, and 1000 μM) in large striatal neurons (presumably LCIs) from WT and symptomatic R6/2 mice. ***B***, Graph shows no significant differences in peak current density (peak current divided by cell capacitance) of GABA responses. ***C***, ***D***, AMPA- and NMDA-induced peak current densities also were similar in R6/2 and WT mice.

To more directly evaluate postsynaptic mechanisms, GABA_A_ receptor-mediated currents were examined in the acutely isolated LCIs in R6/2 mice (*n* = 5, age 71 ± 4 d) and WT littermates (*n* = 6, age 69 ± 3 d). Large cells, presumably LCIs, were identified based on somatic size as well as thick proximal dendritic processes. A range of concentrations (10, 100, 1000 μM) was used to examine the effects of GABA application. GABA-induced peak current densities were not significantly different in LCIs from R6/2 compared to WT mice (Fig. 7*A*,*B*).

AMPA- and NMDA-mediated currents also were assessed in the acutely isolated striatal neuronal preparation, using Mg^2+^-free recording solution. AMPA-induced peak current densities were similar in large cells from WT and R6/2 mice (Fig. 7*C*). NMDA-induced peak current densities also were similar in large cells from WT and R6/2 mice at a range of concentrations (Fig. 7*D*).

## Discussion

The present findings indicate that both morphological and electrophysiological alterations occur in LCIs from symptomatic R6/2 mice. These changes occur in parallel to the reduced capacity for ACh synthesis/release (Vetter et al., 2003; Smith et al., 2006; Farrar et al., 2011). Morphologically, somatic areas of LCIs were smaller than those of WT littermates. In agreement with this, the cell membrane capacitance was significantly decreased while the input resistance was significantly increased in LCIs from R6/2 mice. Although the mean spontaneous firing frequency of LCIs was not altered in R6/2 mice, the firing patterns were significantly affected. More LCIs from R6/2 mice displayed irregular firing and bursts compared to WT littermates, suggesting that the regular, tonic firing ability of LCIs is disrupted in HD. One possible reason firing patterns are altered is that, similar to MSNs, striatal LCIs from symptomatic R6/2 mice show increased GABAergic synaptic inhibition. Optogenetic studies indicated that a likely source of increased inhibition is the SOM-, but not the PV-, expressing interneurons. Altered firing patterns, in conjunction with increased GABA inhibition and reduced somatic size, could partially explain decreased production and release of ACh in HD.

Striatal ACh plays a pivotal role in attention and cognitive processes (Graybiel, 1990, 1995; Ding et al., 2010). ACh synthesis and release are significantly altered in a number of neurological diseases (Bird, 1980; Pisani et al., 2007). For example, Parkinson’s disease and dystonia have been classically considered as hypercholinergic disorders (Pisani et al., 2007). In agreement, ablation of LCIs attenuates l-DOPA-induced dyskinesia in mice rendered parkinsonian (Won et al., 2014). In contrast, in HD, decreased production and release of ACh occur (Vetter et al., 2003; Smith et al., 2006; Farrar et al., 2011), which would contribute to behavioral and cognitive deficits. Furthermore, in animal models of HD, LCIs fail to express LTP, which is a requirement for depotentiation of MSNs and behavioral flexibility (Picconi et al., 2006). The cause for decreased expression of ACh markers in HD remains unknown. However, the findings presented here provide evidence for potential mechanisms.

While the average firing frequency of LCIs was not different in R6/2 and WT animals, the firing pattern was altered. Specifically, regular firing was less prominent in LCIs from HD mice. In slice studies, different types of LCI firing have been observed, including regular, irregular, and bursting (Bennett and Wilson, 1999). In the present study, similar LCI firing patterns were found, but the proportions were different, with more LCIs from R6/2 mice firing irregularly and in bursts. While spontaneous tonic firing of LCIs has been shown to depend on a combination of Na^+^ and *I_h_* currents, the transition to burst firing depends on small conductance Ca^2+^-activated K^+^ (SK) channels (Bennett et al., 2000). In HD mouse models, a number of K^+^ conductances are deficient (Ariano et al., 2005; Tong et al., 2014). It is thus possible that *I_h_* and/or SK channels are also altered in HD animals, which could explain more irregular firing and bursting compared to WT animals.

Although tonic firing of LCIs largely depends on intrinsic conductances (Bennett and Wilson, 1999; Bennett et al., 2000), synaptic inputs can also modulate LCI firing (Bennett and Wilson, 1998). In fact, one critical issue in trying to determine the cause of altered ACh release in HD is whether or not excitatory and inhibitory inputs onto LCIs are affected (Farrar et al., 2011). Anatomical studies have shown that LCIs receive GABA inputs from both direct and indirect pathway MSNs, as well as from GABAergic interneurons (Gonzales et al., 2013). Here, similar to MSNs (Cepeda et al., 2013), LCIs from symptomatic R6/2 mice received increased GABAergic inputs, which would disrupt regular firing and ACh release. While the exact source(s) of increased GABAergic inputs remains unknown, it is likely that feedforward inhibition has an important role. The fact that in the presence of TTX the increase in GABAergic activity no longer occurred, along with similar GABA responses in WT and R6/2 dissociated LCIs, indicates an action potential-dependent presynaptic mechanism. As in the present recording conditions only PLTS interneurons fire spontaneously, it is likely that these interneurons contribute to increased GABA activity. In support, optogenetic stimulation of SOM-expressing GABA interneurons, but not that of PV-expressing interneurons, produced larger responses in LCIs from R6/2 mice. The cause of increased excitability of PLTS interneurons in HD is uncertain. This class of interneurons is resistant to degeneration and ubiquitinated intranuclear inclusions occur very rarely (Kosinski et al., 1999). However, it has been shown that PLTS interneurons fire at higher rates in HD mouse models and are less subject to inhibitory inputs (Cepeda et al., 2013).

The present findings are in general agreement with anatomical data showing that about 60% of all synaptic inputs to LCIs are GABAergic (Gonzales et al., 2013). About 24% of this innervation is from MSN axon collaterals and only 10% is from striatal and/or pallidal PV-expressing neurons. Indeed, similar to our data, while optogenetic stimulation of PV-expressing interneurons induced reliable GABA synaptic responses in MSNs, such stimulation produced no responses in neighboring LCIs (Szydlowski et al., 2013). This means that the bulk of GABA inputs to LCIs come from SOM- or neuropeptide Y-expressing interneurons. Although a small contribution of MSN input to LCIs cannot be completely ruled out, this possibility is unlikely as MSNs recorded *in vitro* are usually very hyperpolarized and do not fire spontaneous action potentials, even though they are more depolarized in symptomatic animals. Additional studies using D1- and D2-Cre animals could sort this out. Interestingly, there is anatomical evidence that the LCI input from direct-pathway MSNs is more prevalent than that from indirect-pathway MSNs (Martone et al., 1992).

Excitatory synaptic inputs also could affect LCI firing rates. Most glutamatergic inputs come from thalamic nuclei (Lapper and Bolam, 1992), are very sparse (representing only about 13% of all synapses), and are localized to the more distal dendrites (Gonzales et al., 2013). Very low spontaneous glutamatergic synaptic activity onto LCIs was found and it was similar in cells from WT and HD animals. In order to increase glutamate release, 4-AP was bath-applied. No significant difference in average frequency of sEPSCs occurred in the presence of 4-AP, indicating that excitatory inputs, at least *in vitro*, are not a likely source of variation in LCI firing. Consistent with this observation, EPSCs evoked by electrical stimulation within the striatum or by specific optogenetic activation of thalamic inputs produced similar responses in LCIs from WT and R6/2 mice. In addition, postsynaptic responses evoked by glutamate receptor agonists in dissociated LCIs were not different.

Together, these data demonstrate that, although in HD there is no loss of LCIs, morphological and electrophysiological abnormalities occur in symptomatic R6/2 mice. However, how changes in firing patterns translate into decreased cholinergic markers in these mice remains an open question. We can only speculate that in order to maintain adequate ACh levels, tonic pacemaker firing of LCIs is required. Phasic bursting and long pauses may disrupt ACh levels and release. In addition, reduced somatic area and possible axonal branching loss could also contribute to reduced ACh markers. Finally, the presence of mutant huntingtin in LCIs could also affect synthesis, transport, and/or release of ACh in spite of no changes in firing rate.

Disruption of regular firing of LCIs can have profound implications in HD. Studies have shown that ACh, acting on muscarinic receptors in corticostriatal terminals, can regulate excitatory input onto MSNs (Pakhotin and Bracci, 2007; Ding et al., 2010). Dysregulated glutamate release is a key feature in the early stages of HD (Cepeda et al., 2007) and it is likely that altered ACh transmission plays a role. Finding ways to restore normal firing patterns of LCIs and prevent altered glutamate inputs could help in the treatment of behavioral and cognitive deficits in HD.

## Synthesis

The decision was a result of the Reviewing Editor Julie Andersen and the peer reviewers coming together and discussing their recommendations until a consensus was reached. A fact-based synthesis statement explaining their decision and outlining what is needed to prepare a revision is listed below. The following reviewers agreed to reveal their identity: Rita Cowell and Anton Reiner

The reviewers and myself in discussing your manuscript find it of potential interest to eNeuro readership, but feel that it requires revision prior to re-consideration for publication based on the points given below.

Clearly an overall strength of the study is the use of optogenetics to analyze cell-specific contributions to the cholinergic interneuron defects in the R6/2 mouse model that may explain alterations in AcH release and later cell loss. However, additional data collection is required in order to validate the findings. These include demonstration that the cre system is functional for all neuronal subtypes examined in both WT and mutant mice and inclusion of additional cells per group in order to see if statistical significance can be reached (the study is underpowered in terms of sample size). It is suggested that this should be via stereological cell counts rather than single section counts. While it is appreciated that viral injection experiments are challenging to perform, it is difficult to interpret the data without a clear idea of the statistical significance.

There are in addition several important features of experimental design which are missing from the manuscript including the promoter used in the viral construct, specifics of method of light pulse delivery, number and ages of animals used per experiment, whether electrophysiological experiments were performed in a blinded manner, fixation methods, etc.

The manuscript also contains ambiguous language and mislabeling particularly in the Results section which makes the authors' exact meaning unclear.

Lastly, there is a general lack of discussion of potential mechanisms underlying the effects observed. For example, which abnormality results in the observed cholinergic cell loss and how? There is also a lack of full consideration of alternative possibilities. For example, could the increase in inhibitory iPSCs contribute to firing irregularities? What is the specific evidence that GABA input from the MSNs may contribute to observed effects? There is also lack of discussion of how data gleaned in this study may relate to affects in other related models of disease.

### Reviewer 1:

#### GENERAL COMMENTS

1) A particular strength of the study is the use of optogenetics to evaluate the contribution of somatostatin interneurons, parvalbumin interneurons, and thalamic afferents to alterations in cholinergic interneuron properties. However, while the results from the somatostatin-cre optogenetics experiments are rather clear (stimulation of those neurons is sufficient to elicit enhanced postsynaptic GABAergic responses), the lack of effect of parvalbumin interneuron stimulation is not well substantiated. The inclusion and display of negative data is important here, considering the profound feed-forward inhibition these neurons can exert on MSNs (especially considering Cepeda et al. 2013, J Neurosci). As an appropriate contrast to the somatostatin optogenetics data, the parvalbumin interneuron data should be shown, including evidence for YFP in parvalbumin-positive interneurons, especially since a lack of effect of optogenetic stimulation of PV-positive neurons could be explained by inefficiency of cre-mediated recombination and channelrhodopsin expression. The authors should also consider adding more data since it seems that the inclusion of a couple more cells per group could reveal a statistically significant increase in response areas and decay times in the R6/2s. It is also important to document YFP expression in thalamic afferents.
2) The authors are encouraged to think about revising the title and abstract to include references to the potential cellular contributors to cholinergic interneuron dysfunction in the R6/2 mouse, considering that the particular novelty of the study comes from the use of optogenetics to identify the neuronal populations that may contribute to the observed alterations in cholinergic interneuron properties.
3) Is there evidence that channelrhodopsin expression is similar between WT and R6/2 groups? It is possible that compromised neurons take up virus more readily, leading to an increased expression of channelrhodopsin and an increase in evoked GABA release from somatostatin interneurons.
4) While an increase in excitatory input onto cholinergic interneurons may not have been predicted, it seems that the authors could increase the sample size even by a small amount and see a significant increase in excitation with optogenetic stimulation of thalamic inputs. The number of cells should be increased for these experiments. In the case that an increase in responses to thalamic terminal excitation is noted, the authors could discuss whether alterations in timing between inhibitory and excitatory stimulation could give rise to the bursting patterns observed in Fig. 2.
5) The authors excessively refer to "trends" that are not statistically significant. While trends can often be used to support an argument in a discussion section, it does not seem appropriate to state trends in results and methods sections (see lines 292, 328, 367, 407, 431, 571, 577). If a trend is interesting enough to warrant comment in the results section, it seems prudent to add additional data points to achieve statistical significance.

#### METHODS

1) Since Sst-cre and PV-cre mice are crossed to R6/2 mice, it is presumed that the channelrhodopsin virus used for those experiments was engineered with a floxed stop codon. Also, it is not stated what promoter was used in that virus - please clarify.
2) Where were light pulses delivered in the thalamic afferent experiments? In the striatum? Was it presumed that local thalamic terminals were activated by the light pulses?
3) How many slices were included in the analysis of mean striatal area for figure 1?
4) It should be stated somewhere how many animals were used for each experiment; the graphs include "n's" but it is assumed that those refer to cells.

#### DISCUSSION

1) What evidence is there that somatostatin interneurons are affected in this model? What could account for increased release of GABA from this population? Is it possible that the number of somatostatin-positive terminals are increased onto cholinergic interneurons for structural reasons (just due to a decrease in neuropil)?
2) How does this data fit into what is known about cholinergic tone in other neurodegenerative disorders, specifically Parkinson's Disease and dystonia? How can the reported changes in firing patters result in the decreased cholinergic tone and behavioral alterations observed in these mice?
3) On line 477, the authors comment that cholinergic interneurons can also receive "GABA inputs from both direct and indirect pathway MSNs" - what is the evidence for or against the possible contribution of MSN-derived GABA to the alterations presented here?

### Reviewer 2 details:

This study uses a combination of anatomy and physiology (more so the latter) to show that although striatal cholinergic interneuron numbers are preserved at about 11 weeks in R62 mice, their electrophysiology is abnormal. Their firing is more irregular, and they show more and larger spontaneous IPSCs. The authors suggest that the abnormalities in cholinergic interneurons found in R62 mice may explain the abnormalities in cholinergic markers and acetylcholine release seen in HD and animal models of HD. The topic is interesting and important. Although the paper presents a convincing case for electrophysiological abnormalities in cholinergic interneurons in R62 mice at about 11 weeks, there are some weaknesses and oversights that would be useful to address.

The tissue fixation for the morphological studies is not mentioned. Clearly the tissue had to be fixed, as free-floating sections were processed immunohistochemically.

The animals used for anatomy are said to be 65 days old in the Methods section, but in the Results section they are said to be about 65 days old. This inconsistency needs to be rectified, and the reader needs to be told the age range and mean age of the cases studied. This also pertains to the electrophysiological studies. These are said (at least in some cases, in others the reader must assume 65 days from context) to be performed at 21 and 65 days, but the age inconsistency in anatomical data raises the concern that the animals analyzed by electrophysiological may not always have been exactly 21 or 65 days old. This matters, as the R62 phenotype is rapidly progressing, and thus cholinergic neurons may change their traits quickly over time.

The authors do not provide stereological cell counts, or even counts for the entire striatum, of cholinergic interneurons. They seem to have the material and data on hand to do so. It would be better to have a count for the whole striatum than to have the single-section counts and cell density numbers.

Anatomy is said to be performed in a blinded manner. Was electrophysiology performed blinded? The number for electrophysiology is presented as cells. How many animals did the cells come from for a particular analysis?

There tends to be problems with the electrophysiological data in how the authors use their statistics. In many types of data, they state that the R62 mean is more (or less) than the WT mean, but the difference is not statistically significant. But then they should not say that the R62 mean was more (or less, as the case may be). That is the whole point of statistics - to determine which differences can be treated as real and meaningful. This circumstance seems to arise because the authors think the outcome should go a certain way, and thus they somehow discount or underplay the importance of the significance. If this is the case, then the authors need to perform more studies to boost the numbers for a given analysis, so the plotted data match the statistical outcome. As it is, I can understand the quandary of the authors. The means do look different in many cases when statistics says no. In these instances, the study may be underpowered, and a clear outcome would perhaps be favored by more analysis. So, while the breadth and diversity of the electrophysiology is impressive, some of it is undermined by the small number of cells analyzed (for example the FSI optogenetics).

For some of the electrophysiological Results, the wording is confusing. For example, line 302 says that 2x as many WT cells as R62 cells displayed "higher autocorrelations". Higher than what? I believe the authors mean simply that 2x as many WT cells as R62 cells displayed high autocorrelations. This then, however, leaves undefined what is meant by high autocorrelation, and why this standard is chosen (whatever it may be).

The authors need to clarify what they mean by the "amplitude of the first lag period" on line 305. I think they mean the amplitude of the response after the immediately prior, and do not mean the amplitude of the inter-spike interval itself. Or perhaps they mean both response and interval.

Line 311 - the authors say that more cells from WT mice than R62 showed a "reduced coefficient of variation". They mean "low" rather than "reduced". Plus, it would be better to first state the criterion of 0.2 for low coefficient of variation, then next state the conclusion. And why is this the criterion anyway?

Lines 319-322 - why should coefficient of variation and firing frequency be related? Why should high firing frequency be linked to low variation in firing rate?

Lines 328-330 - "At 21 days the frequency of sIPSCs was lower in LCIs from R6/2 compared to WT mice but the difference in mean values did not reach statistical significance". This is the type of ambiguous phrasing I noted above. Is there a trend (p=0.42 says no), is the test underpowered, or should we just say they were not significantly different?

Lines 348-349 - "Similar to results obtained with Cs-meth solution, the frequency of mIPSCs recorded after TTX were not significantly different with CsCl". Unclear if WT and R62 are being compared, or Cs-meth and CsCl. Certainly, the former, but still unclear phrasing.

Lines 363-364. Ambiguous, because the qualifier "with shortest latency" is unclear. Presumably, the authors are trying to focus on monosynaptic responses (i.e. with shortest latency).

Some of the analysis is clearly underpowered and leads to no certain conclusion - big differences in means and big error bars - is there or is there not a difference? These instances could be dropped, if the authors do not want to do more studies. For example, the PPR data on lines 365-371. There is no significant difference between WT and R2 cholinergic interneurons in GABA input PPR, the authors say. Nonetheless, the authors invoke a trend, and declare the results as "indicating" that the probability of GABA release onto cholinergic interneurons is higher for R62. The authors need to drop such claims that do not fit the statistics, or perform more studies. Similarly, the FSI optogenetic data.

Line 380 - clearer to say "SOM neuron activation" than 'light stimulation", in my view.

There needs to be some consideration for how putting the R62 transgene on the SOM-cre and PARV-cre backgrounds might affect the cholinergic neuron phenotype? Also, at what age were these studies done?

With regard to the AMPA and NMDA responsiveness of R62 cholinergic neurons to thalamic input activation (by optogenetics), the authors state there is a trend for increased AMPA receptor mediated responses but not for NMDA receptor mediated responses. Yet the plotted data for the two look much the same (increased AMPA and NMDA responses), and we are not presented any actual statistical outcomes in any case.

The histograms in figure 6 identify the mutant mice as BHD150, rather than R62. Are these BHD150 mouse data, or has the R62 data been simply mis-labeled? Also, big error bars for these data, so they are hard to interpret in favor of the null hypothesis. Is this analysis underpowered?

Paragraph beginning line 488. Note that abnormalities in thalamostriatal neuron activity in vivo that are not replicable in vitro could contribute to abnormal cholinergic neuron behavior. For example, if thalamostriatal neurons fire less. Also, in the last sentence the authors conclude there is no differential cholinergic neuron AMPA receptor or NMDA receptor responsiveness to thalamic input between WT and R62 mice. Yet, in the Results section, the authors pointed to a trend for AMPA. Which is it?

The authors broadly conclude that the loss of cholinergic markers in cholinergic neurons in HD may reflect the morphological and electrophysiological abnormalities they find. But which morphological or electrophysiological abnormality would cause a particular cholinergic neuron marker loss. The cholinergic neurons are not less active, as revealed by their spontaneous activity. Moreover, they seem to not differ much in their response to excitatory input.

Finally, is it possible that the increase in spontaneous inhibitory IPSCs in cholinergic neurons in R62 mice might contribute to their firing irregularity? They do not seem to cause a decrease in overall firing rate. Were the cholinergic neurons in R62 mice with the most prominent spontaneous inhibitory IPSCs also those that fired more irregularly?
